# Analytical approaches to evaluate risk factors of multimorbidity: a systematic scoping review protocol

**DOI:** 10.1136/bmjopen-2023-083278

**Published:** 2025-01-28

**Authors:** Wenbo Song, Nick Birk, Mika Matsuzaki, Judith Lieber, Hirotomo Yamanashi, Elliott Rogers, Chanchanok Aramrat, Nutchar Wiwatkunupakarn, Chaisiri Angkurawaranon, Alex Lewin, Sanjay Kinra, Poppy Alice Carson Mallinson

**Affiliations:** 1 London School of Hygiene & Tropical Medicine, London, UK; 2 Nagasaki University, Nagasaki, Japan; 3 Harvard University, Cambridge, Massachusetts, USA; 4 Johns Hopkins University, Baltimore, Maryland, USA; 5 University College London, London, UK; 6 Chiang Mai University, Chiang Mai, Thailand

**Keywords:** Chronic Disease, Risk Factors, GENERAL MEDICINE (see Internal Medicine), GERIATRIC MEDICINE, Multimorbidity

## Abstract

**Abstract:**

**Introduction:**

Understanding causal risk factors that contribute to the development of multimorbidity is essential for designing and targeting effective preventive strategies. Despite a large body of research in this field, there has been little critical discussion about the appropriateness of the various analytical approaches used. This proposed scoping review aims to summarise and appraise the analytical approaches used in the published literature that evaluated risk factors of multimorbidity and to provide guidance for researchers conducting analyses in this field.

**Methods and analysis:**

We will systematically search three electronic databases—Embase, Global Health and MEDLINE, as well as the reference lists of identified relevant review articles, from inception to September 2024. We will screen titles and abstracts using the artificial intelligence-aided software ASReview, followed by screening for eligible articles in full text and extracting data. We will then categorise the analytical approaches used across studies, provide a comprehensive overview of the methodology and discuss the potential strengths and limitations of each analytical approach.

**Ethics and dissemination:**

We will undertake a secondary analysis of published literature; therefore, ethical approval is not required. The results will be disseminated through an open-access, peer-reviewed publication. This systematic scoping review will serve as a guide for researchers in selecting analytical approaches for aetiological multimorbidity research, thereby improving the quality and comparability of research in this field.

**STRENGTHS AND LIMITATIONS OF THIS STUDY**

- Our comprehensive search strategy will identify published literature examining risk factors for multimorbidity in order to summarise and appraise the analytical approaches used across these studies.
- The review protocol was informed by the rigorous and established framework developed by Arksey and O’Malley and adheres to the Preferred Reporting Items for Systematic Reviews and Meta-Analyses extension for Scoping Reviews.
- The two-stage extraction process will facilitate both broader insight into the analytical approaches used across published literature and an in-depth analysis of the appropriateness and relative advantages of each type of analytical approach.
- This systematic scoping review is limited to articles published in the English language.
- This systematic scoping review will not assess the quality of the included articles.

## Introduction

 Multimorbidity is defined as the coexistence of two or more chronic conditions.^1^ It directly affects individual well-being, quality of life and physical functioning, while also placing a significant burden on health system resources and the broader economy.^2 3^ Growing global recognition of multimorbidity as a health concern over the past decade has led to a large body of published research on underlying risk factors for multimorbidity, seeking to understand its causes.^4^,^7^ Evaluating risk factors of multimorbidity is essential for informing health policies and interventions to tackle the rising rates of multimorbidity.^8^,^11^

To date, no consensus has been established on how to evaluate risk factors for multimorbidity, meaning that researchers adopt a wide range of analytical approaches in their studies, some of which may be inappropriate.^12^,^14^ The complex and varied mechanisms underlying multimorbidity present challenges for analysing risk factors and interpreting the results of these analyses.^15 16^ Our preliminary review of the existing literature revealed a large number of studies with a cross-sectional design and evaluating multimorbidity as a single binary outcome using logistic regression. These studies are limited in their ability to infer causality, often lack a defined causal framework or research question and do not attempt to understand whether risk factors contribute to multimorbidity beyond their expected associations with individual conditions. Therefore, recent years have seen increasing concerns about the interpretability and value of their findings.^17^,^20^

The diverse analytical approaches used in existing studies complicate the comparison and synthesis of findings. Age remains the only established risk factor associated with multimorbidity across different populations and contexts.^21 22^ Studies examining other sociodemographic risk factors, such as gender and socioeconomic status, have yielded inconsistent results.^6 20 23^ The extent to which these inconsistencies may be due to varied analytical approaches is unclear. Meanwhile, due to the prevalent analytical issues mentioned above, most proposed behavioural, environmental and biological risk factors for multimorbidity remain speculative or unexplained.^19 20 24^

Existing reviews have extensively examined the measurement of multimorbidity and the clustering methods used to identify multimorbidity patterns.^1 5 25 26^ However, no review has systematically summarised and appraised the analytical approaches used in risk factor analysis of multimorbidity. While the need for a greater understanding of the risk factors of multimorbidity is widely recognised, analytical challenges continue to hinder research in this area.^27^ Summarising the features of the various analytical approaches being used and appraising their appropriateness could help reduce misuse and misinterpretation of methods, thereby improving the quality and fostering consistency and comparability in future studies.^23 28^

To address this need, we plan to conduct a systematic scoping review of all published literature examining risk factors for multimorbidity to summarise and appraise the analytical approaches used. Specifically, our objectives are to:

1. Describe the frequency of different analytical approaches used in published literature examining the risk factors of multimorbidity, in terms of study designs, outcome measures and analysis methods.
2. Synthesise the range of analytical approaches used in the literature into a novel categorisation illustrated with examples.
3. Appraise the advantages, disadvantages and appropriate use cases for each category of analytical approaches.

The findings of this systematic scoping review will be used to propose recommendations on best practice approaches for future studies, which will help to improve comparability between studies and advance understanding of causal risk factors of multimorbidity.

## Methods and analysis

A systematic scoping review of the published literature will be conducted by applying Arksey and O’Malley’s framework which involves five steps: (1) identifying the research question, (2) identifying relevant studies, (3) study selection, (4) charting the data and (5) collating, summarising and reporting results.^29^ A scoping review is chosen over a systematic review as it is more suited to describing key methodological characteristics of a body of literature (as opposed to synthesising the findings of the studies, which is the purpose of a systematic review). The Preferred Reporting Items for Systematic Reviews and Meta-Analyses extension for Scoping Reviews (PRISMA-ScR) checklist will guide the formation and presentation of the scoping review.^30^ The author group of this study includes non-communicable disease epidemiology experts and medical statisticians who will provide advice throughout the scoping review to ensure the quality of study.

### Step 1: identifying the research question

The overall research question for this systematic scoping review is: ‘Which analytical approaches have been used by existing studies to evaluate the risk factors of multimorbidity?’. We define multimorbidity as ‘The co-occurrence of two or more chronic conditions in one person’, as proposed by the WHO. While various definitions of multimorbidity exist, adopting this broad definition allows us to be inclusive of studies which adopt more specific definitions.^31^,^33^ We define risk factors as presumed or putative causal factors contributing to the development of multimorbidity.^7 19^

### Step 2: identifying relevant studies

We will review studies with a primary objective of understanding the risk factors (also termed causes or determinants) of multimorbidity via a systematic search of three major electronic bibliographic databases: Embase, Global Health and MEDLINE, as well as the reference lists of identified relevant review articles. We will consider studies published from inception to 30 September 2024.

We conducted a crude search and found more than 50 studies in this research field, suggesting that there will be sufficient studies to perform this scoping review.^5^ We developed a Boolean search strategy iteratively (see online supplemental appendix A), which was also reviewed by a medical librarian. The search strategy was formulated based on the key terms ‘risk factor’ and ‘multimorbidity’, along with their synonyms. The search strategy will be run across all databases simultaneously through the OVID search interface.

### Step 3: study selection

**Figure 1:**
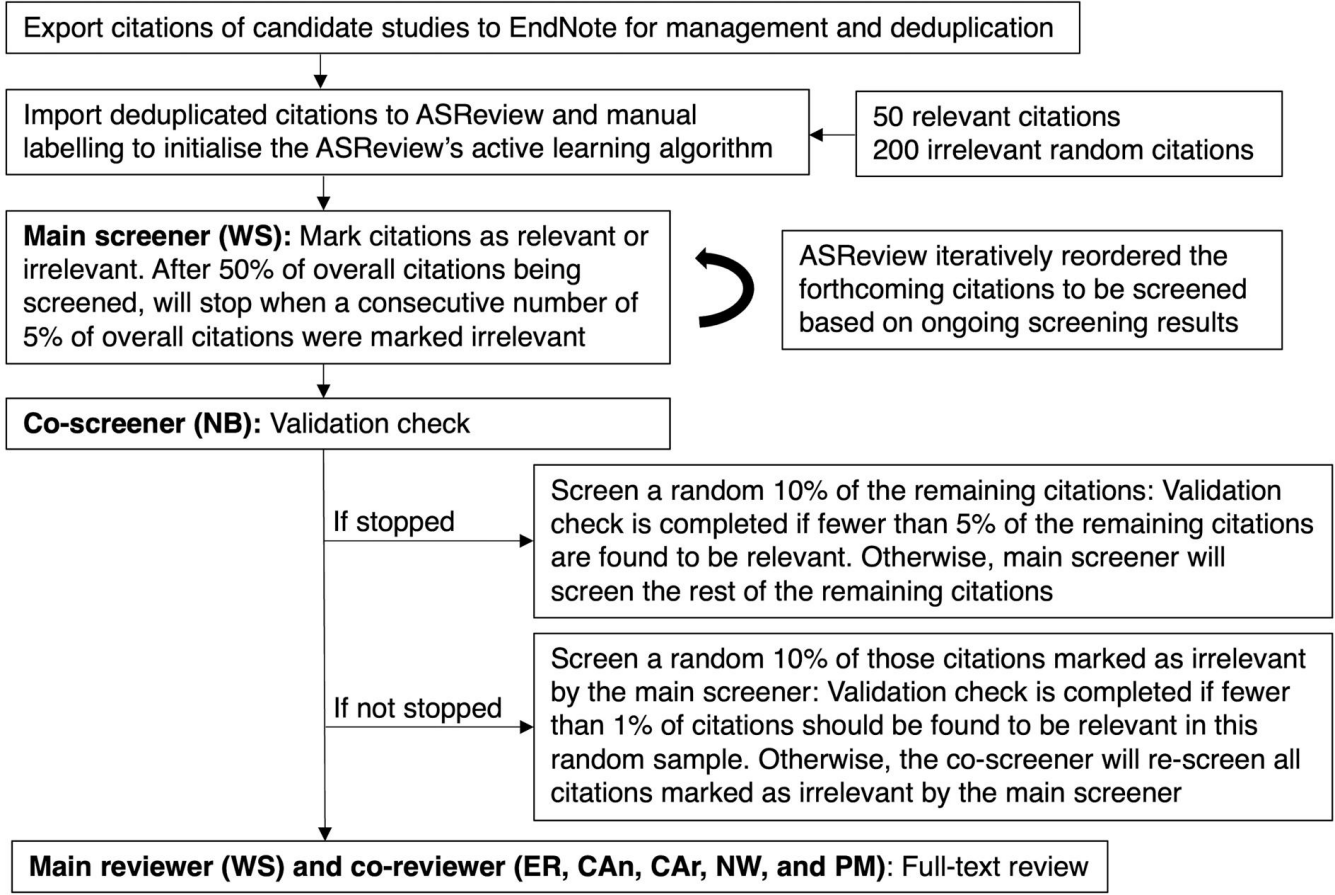
Flow chart of the study selection process.

The flow chart of the study selection process is shown in figure 1. We will export the initial set of candidate citations identified from the electronic bibliographic databases to EndNote 20 bibliographic software for management and deduplication. Subsequently, we will perform title and abstract review, followed by full-text review.

#### Title and abstract review using ASReview

The deduplicated citations will be exported from EndNote and imported into ASReview, an artificial intelligence-aided systematic review software, to screen titles and abstracts against the eligibility (inclusion and exclusion) criteria outlined in table 1. ASReview incorporates an active machine learning technique that iteratively rearranges the citations so that the most relevant are brought to the top of the screen list. This enhances the efficiency of human checking by prioritising the most relevant citations and allowing for the incorporation of stopping rules to complete screening if no further relevant citations are identified, while maintaining transparency throughout the process.^34^

**Table 1:** Inclusion and exclusion criteria

Category	Inclusion criteria	Exclusion criteria
Study design	Quantitative epidemiological studies evaluating any risk factors of multimorbidity conducted in any setting	Abstracts, non-epidemiological studies, qualitative research and case series Studies with sample size smaller than 50
Aim	Studies where a main aim or intention is to understand risk factors of multimorbidity Studies that analyse individual or groups of variables in relation to multimorbidity in an analytical manner, implied by presenting adjusted measures of effects or causal attribution	Studies where a main aim or intention is not to understand risk factors of multimorbidity (eg, descriptive studies)
Population	Adult humans (≥18 years old)	Infants, children or adolescents (<18 years old) Animal research
Exposures	Epidemiological studies examining any risk factors for multimorbidity	Laboratory studies assessing links between biological processes, genomic markers, or genetic traits and multimorbidity
Outcomes	Studies examining, assessing or reporting at least two types of chronic conditions without specific focus on an index condition (to distinguish the concept of multimorbidity from the concept of comorbidity)	Studies with specific focus on comorbidity or complications of specified index conditions
Analytical methods	Quantitative, adjusted approaches models (or with causal attribution between risk factor and multimorbidity implied in another way)	Descriptive statistics only (eg, prevalence of multimorbidity in certain age or sex group)
Publication type	Full-text published article Accessible in English	Conference proceedings, dissertations/theses, editorials/commentaries/letters or articles that do not provide a full-text version Not accessible in English

We will manually label at least 50 relevant citations identified from the reference list of an existing systematic review on multimorbidity,^5^ as well as 200 irrelevant citations randomly generated by ASReview, in order to train the algorithm to initialise the ASReview screening process. The stopping rule of screening for the main screener (WS) is when a consecutive number of 5% of overall citations are marked irrelevant while more than 50% of overall citations have been screened, as proposed by Haastrecht *et al*.^35^ To further confirm that all relevant citations have been identified, we will undertake the following validation checks:

- If the stopping rule is satisfied, the coscreener (NB) will screen a random 10% of the remaining citations (ie, citations not yet screened by the main screener). Fewer than 5% of the remaining citations should be found to be relevant to confirm that at least 95% of eligible citations have been found. Otherwise, the main screener will screen the rest of the remaining citations.
- If the stopping rule is not satisfied, the coscreener will screen a random 10% of those citations marked as irrelevant by the main screener. Fewer than 1% of citations should be found to be relevant in this random sample to confirm the screening validation. Otherwise, the coscreener will rescreen all citations marked as irrelevant by the main screener.

#### Full-text review

After screening the title and abstract, the full texts of selected citations will be further assessed in detail against the same eligibility criteria, as described in table 1. The main reviewer (WS) and coreviewers (ER, CAn, CAr, NW and PACM) will independently review the full texts of each citation with cross-checks performed to ensure consistency. Any discrepancies will be reconciled through discussion between the reviewers, with arbitration resolved by one of the senior investigators (HY and SK).

### Step 4: charting the data

The data extraction will comprise two stages: (1) We will collect and report all studies reporting risk factor analysis for multimorbidity in order to assess the frequency by which different analytical approaches were used in existing studies to date; (2) Given the expected large number of eligible studies, we will provide a comprehensive overview of the methodology of a random subset of five studies from each type of analytical approaches, with particular focus on causal interpretation.

#### Data extraction stage I

We will extract the analytical approaches from eligible studies, focusing on study designs (eg, cross-sectional, longitudinal, case-control), outcome measures of multimorbidity (eg, binary, count, specific cluster) and analytical methods (eg, standard regression modelling, structural equation modelling, network analysis). These analytical approaches will then be categorised. The information extracted will be tabulated to describe the number and percentage of published studies in each category of analytical approaches (see online supplemental appendix B).

#### Data extraction stage II

We will select a random sample (using R software) of five studies from each category of analytical approaches for in-depth data extraction. This number has been chosen in order to illustrate a range of examples from each category, rather than to be exhaustive, to help the reader gain a clearer understanding of what each category involves and inform the discussion of their strengths and limitations. In-depth data extraction from the selected studies will be conducted using a predesigned data extraction table (see online supplemental appendix C), which includes the items listed in table 2 (items may be further developed based on the features of the studies identified).

**Table 2:** Data extraction items (Stage II)

Level	Sublevel/example
Study info	Author’s name and year of study Study title
Study setting	Eg, population (sample size and characteristic), year(s) and duration of follow-up(s)
Study source	Eg, electronic health record, self-report survey or interview, administrative data, clinical routine data, claims data
Study design	Eg, cross-sectional, longitudinal, case-control
Data type	Chronic conditions Number Name Disease identification strategy (eg, physician diagnose, self-report, measured) Coding of conditions (eg, common name of disease, International Classification of Diseases 10th Revision, organ system) Risk factors Number Name
Covariates	
Outcome measure of multimorbidity	Eg, binary or count, patterns of coexisting conditions, weighting of conditions, occurrence order of conditions considered
Analytical method to the risk factor analysis	Main analytical method Main comparison (eg, 0–1 condition vs 2 or more conditions, 0 condition vs 1 condition vs 2 or more conditions, 0 condition vs each specific pattern, no pattern vs pattern A vs pattern B vs, ordinal) Confounding or modification accounted Multilevel modelling used Causal diagram used Considered the effect of risk factors contribute to multimorbidity beyond their expected accumulated associations with individual conditions
Summary of main results	Eg, type of estimate (eg, beta coefficient, RR), adjusted estimates of all included risk factors of multimorbidity, and reported confounding variables being adjusted (eg, age)

### Step 5: collating, summarising and reporting results

We will report the study selection process using a PRISMA-ScR flow chart. In data extraction stage I, we will summarise the information of each category of analytical approaches from eligible studies (see online supplemental appendix B) with descriptive statistics (number and percentage) and report a narrative synthesis of the findings. For studies selected for data extraction stage II, we will describe the extracted data in a table format (see online supplemental appendix C) and appraise each category of analytical approaches (see online supplemental appendix D). Each study can be categorised into multiple methodological categories as deemed appropriate.

### Risk of bias assessment

Since this is a systematic scoping review aiming to identify the range of analytical approaches used in the published literature, we will not assess the overall quality or risk of bias of the included studies.

## Ethics and dissemination

To our knowledge, this is the first systematic scoping review to summarise and appraise the analytical approaches used in the published literature evaluating risk factors of multimorbidity. Given that only published journal articles will be included in this scoping review, ethical approval is not required. Mapping the literature on this topic will help to scope current evidence, clarify research concepts, identify knowledge gaps and provide guidance for future researchers. A summary and appraisal of analytical approaches used to evaluate the risk factors of multimorbidity will help to enhance the evidence generation in this field, expediting its effective translation into practice. The findings from this scoping review will highlight and promote the usage of certain appropriate analytical approaches for researchers, with the aim of improving the quality of research in this field as well as producing more comparable estimates. We are not aware of any existing review or review protocol on this topic published or registered on the International Prospective Register of Systematic Reviews (PROSPERO). We believe the proposed scoping review is feasible and timely. The results will be disseminated via submission for publication to an open-access peer-reviewed journal when complete and will be presented at relevant academic conferences.

## Strengths and limitations

Our study endeavours to identify all published English language literature evaluating risk factors of multimorbidity to provide a comprehensive overview of the analytical approaches used in these studies. The review protocol was informed by the rigorous and established framework developed by Arksey and O’Malley and adheres to the PRISMA-ScR. The two-stage extraction process covers both a broad description of analytical approaches used across the published literature and an in-depth discussion of how these analytical approaches can be used appropriately and their strengths and limitations. The independent double extraction of results minimises the risk of human errors and ensures consistency.

As in any bibliographic search, a potential limitation is the omission of relevant articles from our review. Despite attempting to be exhaustive through the use of three major biomedical databases from the Ovid resources (Embase, Global Health and MEDLINE), plus articles in the references of review articles, it remains possible that relevant publications may be missed. We tried to minimise this risk by designing a wide net search strategy, in which we go beyond overly specific terms such as ‘multimorbidity’ and ‘risk factors’ to reduce the chance of systematically missed articles. This review is limited to articles published in English, as it was judged unfeasible for the research team to conduct searches and extract data in other languages. We acknowledge that this may exclude valuable research published in other languages and could affect the comprehensiveness of the review. However, we still anticipate that the major categories of analytical approaches will be captured through our search strategy. Our protocol specifies that we will select a random subset of five articles from each category of analytical approaches for in-depth data extraction. Although it is possible that we could miss some relevant insights, this possibility is considered minimal, as studies using each category of analytical approaches are likely to be similar with respect to the key methodological learnings. Further, we anticipate that for the more advanced or unusual approaches, there will be fewer than five articles per approach, resulting in the extraction of all these studies. We are not planning to conduct a general quality assessment of included studies, as this would not contribute to achievin our review objectives, which are focused specifically on analytical approaches used to evaluate risk factors of multimorbidity.

## supplementary material

10.1136/bmjopen-2023-083278 online supplemental file 1
