# Supplementary material for: Analytical approaches to evaluate risk factors of multimorbidity: a systematic scoping review protocol
Source: BMJ Open. 2025 Jan 28;15(1):e083278. doi: 10.1136/bmjopen-2023-083278 (PMC11781107; doi:10.1136/bmjopen-2023-083278)
Supplement: online supplemental file 1 [file bmjopen-15-1-s001.docx]

**Supplemental material**

**Appendix A.** Search strategy.

| **Database** | **Tentative title** | **#** | **Search term** |
| --- | --- | --- | --- |
| Embase,  Global Health,  and MEDLINE | multimorbidity | 1 | *Multimorbidity/ or *Multiple Chronic Conditions/ or (multimorbidit$ or multi-morbidit$ or comorbidit$ or co-morbidit$ or polymorbidit$ or poly-morbidit$ or multicondition$ or multicondition$ or "multiple chronic condition$" or "morbidity burden" or ((multiple or coexisting or co-existing or concurrent or con-current or comorbid or co-morbid) adj2 (disease$ or illness$ or condition$ or diagnos$ or morbid$))).ti. |
|  | risk factors | 2 | (*Risk Factors/ or Socioeconomic Factors/ or Life Style/ or Health Behavior/ or Healthy Lifestyle/ or Environmental Exposure/ or (risk factor? or predictor? or determinant? or protective factor? or correlate? or cause? or causa$ or aetiolog$ or etiolog$ or lifestyle factor? or behavioural factor? or behavioral factor? or environmental factor? or demographic factor? or social factor? or socioeconomic factor?).tw.)  not (  *Cells/ or *Cell Biology/ or *Genes/ or *Genome/ or *Bacteria/ or *DNA/ or *Parasites/ or *Microbiology/ or *Vaccines/ or *Pharmacy/ or *Laboratories/ or *"Costs and Cost Analysis"/ or *"Cost of Illness"/ or *Therapeutics/ or *"Allergy and Immunology"/ or *Virology/ or *Viruses/ or *COVID-19/ or (cell$ or gene$ or genom$ or bacteri$ or molecul$ or DNA? or parasit$ or microbi$ or agent? or vector$ or vaccin$ or safety or immun$ or viro$ or viru$ or COVID-19).ti.  ) |
|  | multimorbidity AND risk factors + filter (human, English) | 3 | 1 AND 2 + filter (human, English) |
| $ at the end of a word to indicate truncation; as alternative to “*”  ? inside or at the end of a word to replace zero or one character  .tw. the Text Word (TW) index includes Title (TI) and Abstract (AB); as alternative to “.ti,ab”. | | | |

**Appendix B**. Empty results table for stage I - Categorisation of analytical approaches.

| Analytical approach category | Design | Outcome measure of multimorbidity | Analytical method | *N* | Percentage | References |
| --- | --- | --- | --- | --- | --- | --- |
|  |  |  |  |  |  |  |
|  |  |  |  |  |  |  |
|  |  |  |  |  |  |  |
|  |  |  |  |  |  |  |
|  |  |  |  |  |  |  |

**Appendix C.** Empty results table for stage II - In-depth data extraction of a subset of studies from each category of analytical approaches.

| **Analytical approach category** | **Name and year** | **Setting and source** | **Design** | **Chronic conditions** | | | | **Risk factors** | | **Covariates** | **Outcome measure of multimorbidity** | **Analytical method to the risk factor analysis** | | | | | | **Main results summary** |
| --- | --- | --- | --- | --- | --- | --- | --- | --- | --- | --- | --- | --- | --- | --- | --- | --- | --- | --- |
|  |  |  |  |  |  |  |  |  |  |  |  | **Main analytical method** | **Main comparison** | **Confounding or modification** | **Multilevel modelling** | **Causal diagram** | **Associations beyond simple accumulation accounted** |  |
|  |  |  |  | ***N*** | **Name** | **Disease identification strategy** | **Coding** | ***N*** | ***Name*** |  |  |  |  |  |  |  |  |  |
|  |  |  |  |  |  |  |  |  |  |  |  |  |  |  |  |  |  |  |
|  |  |  |  |  |  |  |  |  |  |  |  |  |  |  |  |  |  |  |
|  |  |  |  |  |  |  |  |  |  |  |  |  |  |  |  |  |  |  |
|  |  |  |  |  |  |  |  |  |  |  |  |  |  |  |  |  |  |  |
|  |  |  |  |  |  |  |  |  |  |  |  |  |  |  |  |  |  |  |

**Appendix D.** Empty results table for appraisal of each category of analytical approaches.

| Analytical approach category | Advantages | Disadvantages | Recommendations |
| --- | --- | --- | --- |
|  |  |  |  |
|  |  |  |  |
|  |  |  |  |
|  |  |  |  |
|  |  |  |  |
