# Supplementary material for: Analytical approaches to evaluate risk factors of multimorbidity: a systematic scoping review protocol
Source: BMJ Open. 2025 Jan 28;15(1):e083278. doi: 10.1136/bmjopen-2023-083278 (PMC11781107; doi:10.1136/bmjopen-2023-083278)
Supplement: Supplementary file 2 [file bmjopen-2023-083278.reviewer_comments.pdf]

# PEER REVIEW HISTORY

BMJ Open publishes all reviews undertaken for accepted manuscripts. Reviewers are asked to complete a checklist review form and are provided with free text boxes to elaborate on their assessment. These free text comments are reproduced below.

## ARTICLE DETAILS

### Title (Provisional)

Analytical approaches to evaluate risk factors of multimorbidity: A systematic scoping review protocol

### Authors

Song, Wenbo; Birk, Nick; Matsuzaki, Mika; Lieber, Judith; Yamanashi, Hirotomo; Rogers, Elliott; Aramrat, Chanchanok; Wiwatkunupakarn, Nutchar; Angkurawaranon, Chaisiri; Lewin, Alex; Kinra, Sanjay; Mallinson, Poppy Alice Carson

---

## VERSION 1 - REVIEW

---

**Reviewer** 1

**Name** Violán, Concepción

**Affiliation** Institut Universitari d'Investigació en Atenció Primària Jordi Gol (IDIAP Jordi Gol)

**Date** 18-Feb-2024

**COI** NO

---

Page 8, lines 184-7 The author's comment

“On data extraction stage II, a random sample of up to 5 studies from each analytical approach 185 categories will be selected for in-depth data extraction to inform the appraisal of the approaches using the pre-designed master data extraction table (see Appendix C), which includes the below (and may be developed further based on features of the studies identified)”.

The decision to use only 5 articles for each analytical approach to conduct a thorough review could be appropriate since it is a scoping review. However, the authors should justify why 5 articles were selected and not another number of articles. It is also not clear why they do not propose to review all articles, even if the quality is not evaluated or a risk assessment is not performed.

If not reviewing all articles, discussing a percentage of articles for each method might be better. There may be methods with many articles and methods with few articles, leading to a better analysis of some methods over others.

Figure 1 should have its quality improved; it appears blurry.

Appendix 3 would be better presented in landscape orientation; the font is too small. Despite enlarging the table, it isn't easy to read.

---

| | |
|---|---|
| **Reviewer** | **2** |
| **Name** | **Dasi, Teena** |
| **Affiliation** | **National Institute of Nutrition** |
| **Date** | **28-Feb-2024** |

**COI** **I would like to declare that I have co-authored one paper with a few authors of this manuscript ( https://pubmed.ncbi.nlm.nih.gov/38011977/ BMJ Open. 2023 Nov 27;13(11):e073897. Doi: 10.1136/bmjopen-2023-073897. PMID: 38011977; PMCID: PMC10685937.)**

---

The introduction clearly sets the context and highlights the importance of understanding analytical approaches in multimorbidity research. The methods section is well-structured and detailed, for the review process. The inclusion of an author group with expertise in different areas strengthens the review's credibility. Overall, this is a well-designed and well-presented scoping review protocol.

However, few comments given here could be addressed to further enhance the comprehensiveness and rigor of the scoping review.

1. In the introduction, you mention studies lacking a defined "causal framework or question." It would be helpful to clarify how you will assess those studies in the review.

2. While the need for a systematic scoping review is justified, it might be beneficial to briefly mention why a scoping review is chosen over a systematic review and how it aligns with the research objectives.

3. Consider revising the objectives section to provide more specific and measurable goals for the study, which could enhance the clarity and focus of the research.

4. In the methods section, you could add a brief explanation of how you will handle studies that use multiple analytical approaches (e.g., will you categorize them into multiple categories or select a primary approach?)

5. In the data extraction stage II, while you plan to select up to 5 studies per analytical approach, providing specific criteria for selection (e.g., diversity, representativeness) would enhance transparency and justification.

6. In the data extraction section, could you add a column to the table in Appendix B to specify the number of studies using each analytical approach for each risk factor category (e.g., socioeconomic, behavioral)

7. In the data extraction stage II, could you consider adding a column to the data extraction table for "Strengths and limitations of the study design and analysis." This would help you to better appraise the different analytical approaches.

8. In the data extraction section, could you clarify what is meant by "special outcome measures of multimorbidity" in Appendix C.

9. Elaborate on the limitations section to discuss potential biases, sources of error, and implications for the interpretation of findings. This could provide valuable insights for researchers and readers.

10. The protocol mentions adherence to PRISMA-ScR guidelines, but there's no specific mention of trial registration, funding or other supplementary reporting details.

---

| | |
|---|---|
| **Reviewer** | **3** |
| **Name** | **Lee, Poay Sian Sabrina** |
| **Affiliation** | **National Healthcare Group Polyclinics, Clinical Research Unit** |
| **Date** | **13-May-2024** |
| **COI** | **Nil** |

---

Choice to use ASReview software to sort articles, compared to conventional Covidence

- What is the accuracy of the software?

Date of search - inception until December 2022

- Should this date be further updated to 2023, since we are looking at May 2024? There would be new articles being published for the entire 2023 which would have been missed out

Boolean strategy

- Was it done with consultation of a medical librarian?

My last question - what is the added value of this scoping review? There are many scoping reviews of multimorbidity out there, what is the novelty here?

---

## VERSION 1 - AUTHOR RESPONSE

**Reviewer 1:**

- **Page 8, lines 184-7 :"On data extraction stage II, a random sample of up to 5 studies from each analytical approach 185 categories will be selected for in-depth data extraction to inform the appraisal of the approaches using the pre-designed master data extraction table (see Appendix C), which includes the below (and may be developed further based on features of the studies identified)". The decision to use only 5 articles for each analytical approach to conduct a thorough review could be appropriate since it is a scoping review. However, the authors should justify why 5 articles were selected and not another number of articles. It is also not clear why they do not propose to review all articles, even if the quality is not evaluated or a risk assessment is not performed.**
    - From known articles and existing review articles, our preliminary search suggests that hundreds of articles could be eligible for this scoping review, and we are likely to categorise them into a dozen categories. We expect studies within each category to be relatively similar with respect to key relevant features of their analytical approaches. Therefore, we do not feel it would add value to extract every study within each category, as instead only a couple would serve to illustrate this point. We specifically chose 5 in order to saturate the methodological approaches whilst capturing the range of approaches taken, and ensuring that the amount of data extraction would be feasible. We have added to the methods section to explain this justification in Lines 236-239: "This number has been chosen to be illustrative of a range of examples from each approach, rather than to be exhaustive, in order to help the reader gain a clearer understanding of what each approach involves and inform discussion of their strengths and limitations".
- **If not reviewing all articles, discussing a percentage of articles for each method might be better. There may be methods with many articles and methods with few articles, leading to a better analysis of some methods over others.**
    - From known articles and existing review articles, we found that a large number of studies used a similar analytical approach of "cross-sectional design, binary outcome of multimorbidity, and binary logistic regression". As reported in Lines 84-91: "Our preliminary review of the existing literature revealed a large number of studies with cross-sectional design evaluating multimorbidity as a single binary outcome using logistic regression. These studies are limited in their ability to infer causality, often lack a defined causal framework or research question, and do not attempt to understand whether risk factors contribute to multimorbidity beyond their expected associations with individual conditions. Therefore, recent years have seen increasing concerns about the meaningfulness and comprehensiveness of their findings". Selecting articles for in-depth review based on the percentage of articles would result in an overemphasis on these studies. Since we would like to equally appraise various analytical approaches regardless of their frequency of usage, with more relative emphasis on newer and more advanced approaches, we will select articles from each analytical approach based on absolute numbers rather than percentages.
- **Figure 1 should have its quality improved; it appears blurry.**
    - Thank you for your suggestion; we have improved the quality of Figure 1.
- **Appendix 3 would be better presented in landscape orientation; the font is too small. Despite enlarging the table, it isn't easy to read.**

- Thank you for your suggestion; we have increased the font size of Appendix C (Appendix 3) to enhance readability.

<u>Reviewer 2:</u>

- **The introduction clearly sets the context and highlights the importance of understanding analytical approaches in multimorbidity research. The methods section is well-structured and detailed, for the review process. The inclusion of an author group with expertise in different areas strengthens the review's credibility. Overall, this is a well-designed and well-presented scoping review protocol. However, few comments given here could be addressed to further enhance the comprehensiveness and rigor of the scoping review.**
  - Thank you for your comment.
- **In the introduction, you mention studies lacking a defined "causal framework or question." It would be helpful to clarify how you will assess those studies in the review.**
  - Understanding causal risk factors contributing to the development of multimorbidity is essential for designing and targeting effective preventive strategies. When assessing analytical approaches in this scoping review, we will specify whether the main analytical method involved a causal inference analytical framework and whether the analysis was informed by a causal diagram (eg, Directed Acyclic Graph) (see Line 244 Table 2 and Appendix C). If applicable, we will discuss the advantages and disadvantages of such approaches for multimorbidity research in the discussion section of the scoping review.
- **While the need for a systematic scoping review is justified, it might be beneficial to briefly mention why a scoping review is chosen over a systematic review and how it aligns with the research objectives.**
  - Thank you for your suggestion. We have added in Lines 134-137 to explain why a scoping review is chosen over a systematic review and how it aligns with the research objectives in the methods section: "A scoping review is chosen over a systematic review as it is more suited to describing key methodological characteristics of a body of literature (as opposed to synthesising the findings of the studies, which is the purpose of a systematic review)".
- **Consider revising the objectives section to provide more specific and measurable goals for the study, which could enhance the clarity and focus of the research.**
  - Thank you for your suggestion. We have amended the objectives section in Lines 111-123 by providing more specific and measurable goals for the study.
- **In the methods section, you could add a brief explanation of how you will handle studies that use multiple analytical approaches (e.g., will you categorize them into multiple categories or select a primary approach?)**
  - Information about handling studies that used multiple analytical approaches is currently contained in the methods section in Lines 298-299: "Each study can be categorised into multiple methodological categories as deemed appropriate".
- **In the data extraction stage II, while you plan to select up to 5 studies per analytical approach, providing specific criteria for selection (e.g., diversity, representativeness) would enhance transparency and justification.**
  - Thank you for your suggestion. We have amended the methods section in Lines 234-236 regarding the specific criteria for selection to clarify that these will be selected at

random: “We will select a random sample (using R software) of 5 studies from each category of analytical approaches for in-depth data extraction”.

- **In the data extraction section, could you add a column to the table in Appendix B to specify the number of studies using each analytical approach for each risk factor category (e.g., socioeconomic, behavioral)**
    - Given that there have been several systematic review articles, such as those by Xu et al., (2017), Tazzeo et al. (2023), and Goet et al. (2024), which summarised the risk factors for multimorbidity, our study places more emphasis on appraising the analytical approaches taken by this body of literature, rather than focusing on which risk factors were evaluated.
- **In the data extraction stage II, could you consider adding a column to the data extraction table for "Strengths and limitations of the study design and analysis." This would help you to better appraise the different analytical approaches.**
    - Thank you for your suggestion. We have added the “Appendix D. Appraisal of each category of analytical approaches” to reflect this recommendation.
- **In the data extraction section, could you clarify what is meant by "special outcome measures of multimorbidity" in Appendix C.**
    - Thank you for your suggestion. We agree this is unclear, so have replaced this with a list of specific ways of defining multimorbidity outcomes instead (e.g. count of chronic conditions, specific pattern, weighting of conditions, etc) (see Table 2 in Line 244).
- **Elaborate on the limitations section to discuss potential biases, sources of error, and implications for the interpretation of findings. This could provide valuable insights for researchers and readers.**
    - Thank you for your suggestion. We have elaborated on the limitations section in Lines 339-364.
- **The protocol mentions adherence to PRISMA-ScR guidelines, but there's no specific mention of trial registration, funding or other supplementary reporting details.**
    - As this is a scoping review, which differs from a systematic review, it does not typically require prior registration. Specifically, PROSPERO does not provide registrations for scoping review. Information about the funding is currently contained in the supplementary information in Lines 379-381.

<u>Reviewer 3:</u>

- **Choice to use ASReview software to sort articles, compared to conventional Covidence: What is the accuracy of the software?**
    - ASReview uses advanced machine learning approaches to reorder the titles and abstracts to prioritise those most likely to be relevant; in this way, it goes beyond the functionality of Covidence to be more intelligent and assist human reviewers in their tasks. As explained by ASReview[1]: “Finding 100% of the relevant papers appears to be almost impossible, even for human annotators. Therefore, we typically aim to find 95% of the inclusions.”. Information about the usage and accuracy of ASReview is currently contained in Lines 179-189: “ASReview incorporates an active machine learning technique that iteratively rearranges the citations so that the most relevant are brought

---

[1] https://asreview.nl/blog/active-learning-explained/

to the top of the screen list. This enhances the efficiency of human checking by prioritising the most relevant citations and allowing for the incorporation of stopping rules to complete screening if no further relevant citations are identified, whilst maintaining transparency throughout the process". We have designed a comprehensive stopping rule for screening to ensure that we do not stop too early and miss potentially relevant papers. This includes screening a random portion of all those titles deemed less relevant by ASReview, and restarting human review if we find relevant papers included there. Based on this, we expect to achieve similar accuracy to conventional screening software, such as Covidence.

- **Date of search - inception until December 2022: Should this date be further updated to 2023, since we are looking at May 2024? There would be new articles being published for the entire 2023 which would have been missed out.**
    - Thank you for your suggestion. See our response to the second suggestion from the Editors.
- **Boolean strategy: Was it done with consultation of a medical librarian?**
    - Yes, we took inputs from a medical librarian at the London School of Hygiene & Tropical Medicine in the development of this boolean search strategy.
- **My last question - what is the added value of this scoping review? There are many scoping reviews of multimorbidity out there, what is the novelty here?**
    - The focus and objectives of our review differ from any other published studies. Despite a number of scoping reviews of multimorbidity, there is little critical discussion around the appropriateness of the different analytical approaches used in analysing behavioural risk factors for multimorbidity. By summarising and appraising the analytical approaches used across these studies, our study will act as a guide for researchers selecting analytical approaches for aetiological multimorbidity research and improving the quality and comparability of research in this field.
